# Examination of the knowledge gap of return-to-work outcomes in routine outpatient treatment for common mental disorders: a systematic review

**DOI:** 10.3389/fpsyg.2023.1167058

**Published:** 2023-11-03

**Authors:** Jakob Lundqvist, Martin Brattmyr, Martin Schevik Lindberg, Audun Havnen, Stian Solem, Odin Hjemdal

**Affiliations:** ^1^Department of Psychology, Norwegian University of Science and Technology, Trondheim, Norway; ^2^Health and Welfare, Trondheim Municipality, Norway; ^3^Nidaros Community Mental Health Centre, Division of Psychiatry, St. Olav’s University Hospital, Trondheim, Norway

**Keywords:** treatment as usual, absenteeism, sick leave, common mental disorders, anxiety, depression

## Abstract

**Objective:**

Little is known about the effects of routine mental health care on return-to-work (RTW) outcomes. This systematic review aimed to summarize and evaluate the effects of clinical representative psychotherapy on RTW among patients with a common mental disorder (CMD), treated within public mental health care.

**Method:**

A systematic search was conducted using PubMed, PsycINFO, Embase, and SveMED+. Primary outcomes were RTW, sick leave status, or self-reported work functioning. Studies limited to specific treatments and/or specific patient groups were excluded.

**Results:**

Out of 1,422 records, only one article met the preregistered inclusion criteria. After broadening of criteria, a total of nine studies were included. Six were randomized controlled trials (RCT), two were register-based studies, and one was a quasi-experimental study. Descriptions of treatment duration and intensity of usual care were rarely specified but ranged from a few sessions to 3 years of psychotherapy. In the RCTs, two studies favored the intervention, one favored routine care, and three found no difference between conditions. Choice of outcomes differed greatly and included RTW rates (full or partial), number of days until RTW, change in sick leave status, and net days/months of work absence. Time points for outcome assessment also varied greatly from 3 months to 5 years after treatment.

**Conclusion:**

There is inconclusive evidence to establish to what extent routine mental healthcare is associated with improved RTW outcomes for patients with CMD. There is a need for more and better clinical trials and naturalistic studies detailing the content of routine treatment and its effect on RTW.

**Systematic review registration:**

This study was pre-registered at PROSPERO (CRD42022304967), https://www.crd.york.ac.uk/prospero/display_record.php?ID=CRD42022304967.

## Introduction

Mental disorders are the largest contributor to the burden of disease worldwide and one of the most common causes of absenteeism (Vigo et al., 2016). These disorders are associated with great personal, social, and financial costs to employees, employers, and society (Hensing and Wahlström, 2004; Harvey et al., 2009). At any given time, one in five persons globally meet diagnostic criteria for anxiety- or depressive disorders, which are frequently labeled as common mental disorders (CMD; Steel et al., 2014). These disorders are often recurrent, with a high risk of relapse after treatment (Koopmans et al., 2011). Sick leave due to CMD is an increasingly recognized societal challenge (Lidwall et al., 2018). Even though CMD is a major cause of absenteeism and sick leave, very little is known about the effect of clinical representative psychotherapy, often labeled treatment as usual (TAU), on return-to-work (RTW).

Typically, TAU implies that patients with mental health disorders receive non-manualized treatment from healthcare professionals in a public mental health clinic (Johnson et al., 2016). The treatment is often adjusted to the patients’ needs, therapist’s preference and seldom systematically assessed. There are clinical guidelines with recommendations for the treatment of CMD, but they are rarely used in ordinary clinical practice (Johnson et al., 2016). In addition, work-related interventions are given less prominence in treatment guidelines compared to interventions aimed at symptom reduction. Furthermore, psychological treatment for mental illness does not necessarily contribute to reduced absenteeism and may not be of clinical significance (Finnes et al., 2019a).

Despite being frequently used as a generic control condition in randomized controlled trials (RCT), the effect of TAU is seldom investigated in detail (Wampold et al., 2011; Kazdin, 2015; Watts et al., 2015). Most RCTs examine the effect of a specific treatment based on research premises, such as experimental procedures and using specially trained clinicians, rather than a clinically representative treatment where patients are referred and often have heterogeneous characteristics (Shadish et al., 1997). The use of ambiguous definitions of TAU, where the content varies and is frequently not described in detail, makes it difficult to generalize and examine the quality and effect of the treatment delivered.

What has been typically considered as TAU varies between countries, regions, clinics, and healthcare providers (Freedland et al., 2011). In previous reviews that focused on CMD and RTW, TAU has varied with respect to healthcare settings, duration of treatment, and type of healthcare professions providing the treatment (Salomonsson et al., 2017; Finnes et al., 2019a; Axén et al., 2020; Nieuwenhuijsen et al., 2020; Christie et al., 2021). Treatment provided by social insurance officers, trade union personnel, and self-help websites, exemplifies definitions of TAU in various studies. Additionally, an immense disparity in treatment duration was noted among patients allocated to the control condition of TAU in the reviewed studies. While some studies provided comprehensive treatment, others provided 10 min appointments with their general physician (GP) primarily for renewal of their sick listing. Some participants were not offered clinical treatment, while some were put on a waiting list. In some studies, patients in TAU conditions were recommended to find help elsewhere, while others were e-mailed information regarding the welfare system. In summary, the research field of TAU is complex due to the large variation in definitions and treatment contents (Wampold et al., 2011; Kazdin, 2015).

Several systematic reviews have addressed the treatment effects of specific interventions on RTW for people with mental health disorders on sick leave, but the effect is ambiguous (Pomaki et al., 2011; Dewa et al., 2015; Perski et al., 2017; Axén et al., 2020). Some studies imply that specific psychological treatment, such as cognitive behavior therapy (CBT) reduces sickness absence in patients not on sick leave at start of treatment (Hägglund et al., 2020), while others argue for no difference between CBT and TAU (Ejeby et al., 2014; Tingulstad et al., 2020). Two recent reviews focused on treatment in various settings for patients with CMD found that psychotherapy had a small effect on RTW compared to TAU (Salomonsson et al., 2017; Finnes et al., 2019a). A related review found no evidence that work-focused interventions alone increased RTW in patients with depression (Nieuwenhuijsen et al., 2020). However, several reviews have indicated that work-focused interventions along with psychological interventions could reduce the time to first RTW (Nigatu et al., 2016; Axén et al., 2020; Nieuwenhuijsen et al., 2020). Yet, the effect of work-focused interventions varies with respect to time to full RTW and the percentage working at follow-up (Joyce et al., 2016; Nigatu et al., 2016; Axén et al., 2020). An estimation found a mean of 165 days (*SD* = 103) until full RTW for those receiving TAU within OHS or primary care (Nigatu et al., 2016). However, these findings might be difficult to generalize to treatment provided within routine care, because of the ambiguous definitions and content of TAU.

It is unclear if TAU in routine care for patients on sick leave due to CMD increases RTW. The present review aims to evaluate if clinical representative treatment for adult outpatients with CMD is associated with improved RTW. This review will add to the empirical literature by not limiting inclusion criteria to specific treatment interventions aimed at increasing RTW. To the best of our knowledge, this is the first review that summarizes the RTW effect of routine treatment for patients with CMD.

## Methods

### Protocol and registration

This systematic review was conducted in line with the PRISMA guidelines (see Supplementary material) and a pre-registered protocol in PROSPERO (CRD42022304967) to identify reports and studies of the RTW-effects of clinical representative TAU for patients on sick leave due to CMD.

### Description of definitions

In this review, CMD was defined as a mix of patients diagnosed with (or had elevated symptoms of) depression, anxiety disorder, insomnia, stress, or burnout defined as parts of adjustment disorders. A broad definition of absenteeism, including both RTW and sickness absence was used as the primary outcome. We assumed a direct relationship between the concepts of RTW and sick absence that implies that a reduction of days of sickness absence increases RTW. RTW was defined as the duration of sick leave in days from the day of randomization until full or first day of RTW or by the proportion of participants that had achieved full RTW at follow-up. Sickness absence was defined as a financially compensated temporary medically certified absence due to any illness or injury. Data regarding absenteeism could either be presented as continuous data (e.g., means, median, or standard deviations of days to RTW) or as categorical data (e.g., the proportion of participants with partial or full RTW at follow-up) from the start of treatment.

We used the term TAU to indicate an active clinic-based outpatient therapy as defined by Shadish et al. (1997). A clinical representative treatment that’s not affected by researchers and is provided by healthcare professionals with a common workload. Hence, TAU was characterized as non-monitored treatments provided to a heterogeneous patient group that has been referred and not recruited to a therapist trained for the study, and where therapists have been able to choose several methods and not usually follow a protocol (Shadish et al., 1997).

### Inclusion and exclusion criteria

Eligible studies used the following criteria: the routine treatment was delivered to employed adult outpatients diagnosed with CMD in routine care. The intervention had to be active and delivered by healthcare professionals within public mental healthcare facilities. The sample should be clinically representative, consisting of a mix of diagnoses, including at least two different common mental disorders, e.g., an anxiety disorder and major depressive disorder. Records had to include outcome measures relating to sick leave levels, including sickness absence, RTW, or self-reported work functioning. All quantitative study designs were allowed. No restrictions were applied regarding country-of-origin or year of publication.

Samples of patients with severe mental health disorders (e.g., schizophrenia) or patients treated at specific treatment units (e.g., acute psychiatric ward) were excluded, as were records limited to specific disorder groups. Studies conducted within the private practice including OHS were not included. Records examining only one specific treatment technique or modality (e.g., CBT, work-focused CBT, internet therapy) were excluded, as were other types of non-routine psychotherapy (i.e., enhanced care, psychological placebo, groups with self-help books, waiting-list groups and non-treatment studies, or only pharmacological treatment).

### Search strategy

An extensive systematic search in PubMed, PsycINFO, Embase, and SveMED+ was conducted. Reference lists of relevant systematic reviews were screened. The reference lists of identified articles were scrutinized and citing articles were identified using Scopus.com, thus backward and forward citation searching was conducted. Publications in English and Scandinavian languages were included. The search was first made on the 26th of January 2022, the second search on the 26th of June 2022 and the final on the 3rd of October 2022. No restrictions were applied with regard to publication dates. The search strategy was first piloted in PubMed and then adjusted for mapping terms in PsycINFO, Embase, and SveMED+. The search string was made of three main concepts: ‘absenteeism,’ ‘mental health’, and ‘treatment as usual.’ The search string is presented in Supplementary material.

### Data extraction process

Duplicates were first discarded in Endnote and then in the web tool Rayyan. Two authors (JL and MB) independently and blinded, screened the title and abstract for eligibility according to the pre-registered PROSPERO protocol (CRD42022304967). The reference list of eligible articles after screening was examined for additional references. Articles eligible for full-text reading were evaluated, and for excluded articles, the first detected criterion for exclusion was reported. Conflicting results were conferred with all authors to reach a consensus.

### Assessment of risk of bias in the included studies

For each RCT publication, two of the authors independently assessed the methodological quality of the included studies using the Cochrane Risk of Bias tool (ROB-2) for RCT (Sterne et al., 2019). Any disagreements between the reviewers were resolved through consensus discussion. ROB-2 assesses six dimensions of bias, including bias arising from the randomization process, bias related to the timing of randomization, bias due to deviations from intended interventions, bias due to missing outcome data, bias in the measurement of outcomes, and bias in the selection of reported results. The included studies were individually assessed within each of these dimensions and categorized as either low risk, some concerns, or high risk.

## Results

### Literature search and study selection

**Figure 1:**
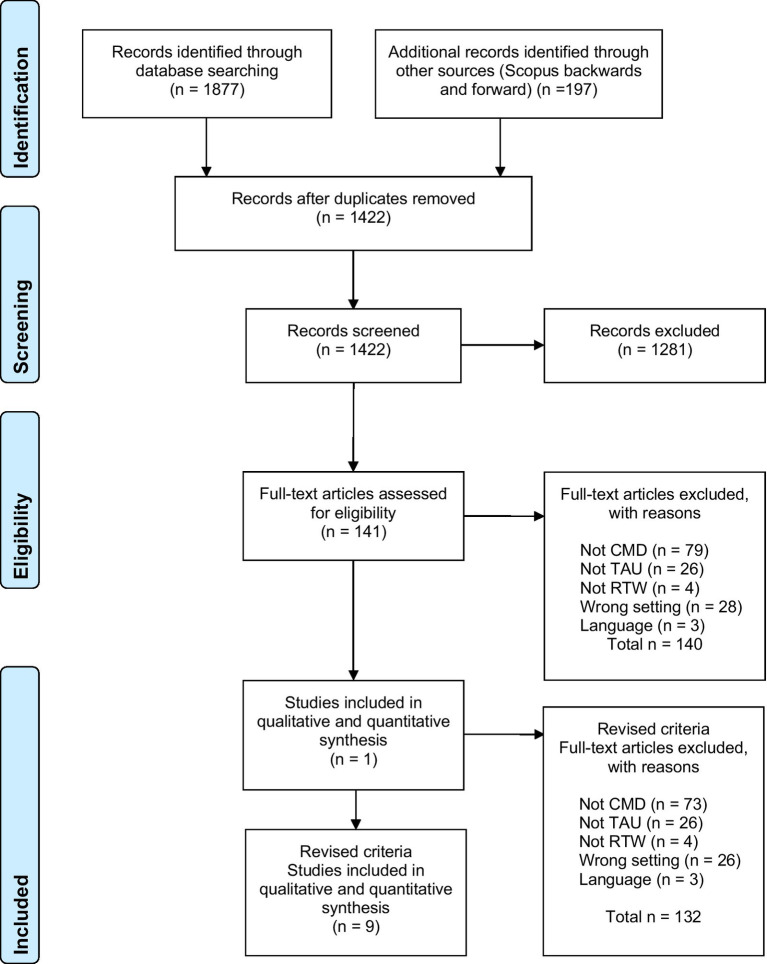
Flow chart of included studies.

The search revealed 1,422 potential records for which abstracts were read. After excluding ineligible records, the full text was retrieved and scrutinized for 141 records. Full-text eligible records were then subsequently examined (see Figure 1).

Only one study met the pre-registered inclusion criteria (Finnes et al., 2019b). We, therefore, decided to broaden the inclusion criteria to include studies restricted to one specific CMD diagnosis and studies with non-manualized psychotherapy provided within OHS. Studies that were limited to specific treatment techniques or modalities, such as work-focused treatment or manual-based CBT only, were not considered as clinically representative treatments and were excluded. When re-examining the excluded records with the revised inclusion and exclusion criteria, eight additional articles were included yielding a total of nine articles eligible for systematic review. Excluded full-text records are presented in Supplementary material.

**Figure 2:**
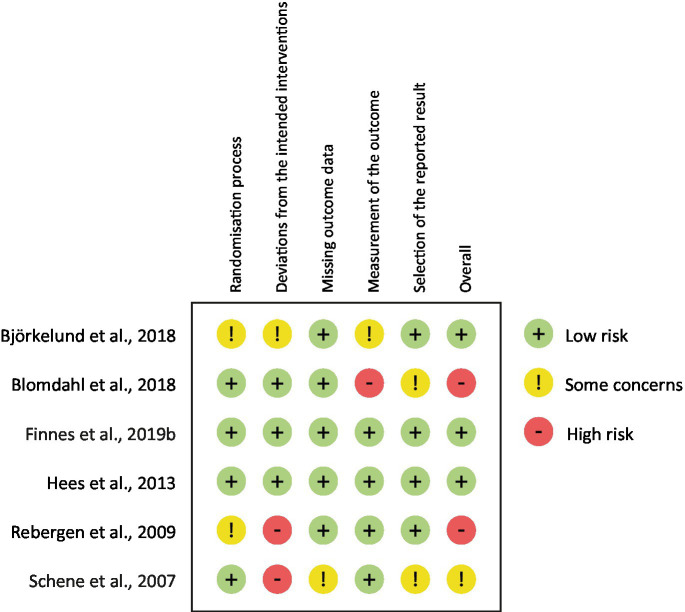
Risk of bias in included studies.

The risk of bias was low for three RCTs (Hees et al., 2012; Björkelund et al., 2018; Finnes et al., 2019b). Some concerns were identified in the assessment of Schene et al. (2006) due to deviations from the intended interventions. Two studies had a high risk of bias due to deviations from the intended interventions (Rebergen et al., 2009) and bias in the measurement of outcomes (Blomdahl et al., 2018). See Figure 2 for further information.

### Systematic review of included studies using revised inclusion and exclusion criteria

Of the nine included studies there were six RCTs, two register-based studies, and one quasi-experimental study (see Table 1). Four studies were from Sweden, three from the Netherlands, one from Canada, and one from Finland. Five of the studies included samples of depressed patients, three had CMD samples, and one study included “stressed” patients (work-related psychological complaints). Sample sizes per condition in the RCTs ranged from 24 to 184, while the register-based studies had large samples with 10–20,000 people. In the RCTs, usual care was compared with different interventions. These included TAU + care managers, TAU + art therapy, Acceptance and Commitment Therapy along with workplace dialogue, adjuvant occupational therapy (two studies), and guideline-based care by occupational physicians. Hence, in many of the RCTs, TAU was compared with TAU plus an additional intervention which probably resulted in uneven amounts of treatment delivered.

**Table 1:** Summary of included studies after broadening of inclusion criteria.

Study	(1) Country (2) Sample	(1) Design (2) TAU (*n*) (3) Comparison group (*n*) (4) Sickness absence at inclusion	(1) Setting (2) Therapists (3) Treatment (4) Duration	Results
Björkelund et al. (2018)	(1) Sweden (2) Depression	(1) RCT (2) *n* = 184 (3) TAU + Care managers (*n* = 192) (4) 55%	(1) Primary care. (2) NA. (3) Swedish guidelines for depression and anxiety: CBT, guided self-help, interpersonal, psychopharmacology. (4) 6–7 contacts across 12 weeks for intervention group. Not specified for TAU.	Intervention group had better return to work rates and lower depression scores at 3–6 months follow-up. Mean number of sick leave days was reduced from 66 to 62 for TAU, and 43% returned partially to work.
Blomdahl et al. (2018)	(1) Sweden (2) Depression	(1) RCT (2) *n* = 36 (3) TAU + Art therapy (*n* = 43) (4) NA	(1) Primary care. (2) Occupational therapists. (3) Acupuncture, CBT, behavioral, ECT, interpersonal, occupational, psychodynamic, pharmacological, physical activity, supportive, and physiotherapy. (4) 10 weekly 1 h sessions for intervention group. Not specified for TAU.	The intervention group had a higher degree of return to work and improvement in depression than TAU at 3-month follow-up. There was no significant change in sick leave (3.4% showed improvement) or depression for TAU.
Ebrahim et al. (2013)	(1) Canada (2) Depression	(1) Register (2) *n* = 20,846 (3) NA (4) 100%	(1) Psychiatric outpatient. (2) NA. (3) Psychotherapy, CBT. (4) Six ACT sessions. For TAU: 51.6% met with a psychologist for a mean of 4.1 sessions.	Receipt of psychotherapy was associated with longer time to short-term disability claim closure and faster long-term disability claim closure. 32.8% with partial RTW for short-term claims and 68.1% for long-term claims.
Finnes et al. (2019b)	(1) Sweden (2) CMD	(1) RCT (2) *n* = 88 (3) Acceptance and commitment therapy (ACT, *n* = 89), workplace dialogue (WDI, *n* = 87), and ACT + WDI (*n* = 88) (4) 42.5% (100% sick leave)	(1) Psychiatric outpatient. (2) GP, psychologist, social worker, physical therapist, nurse. (3) NA. (4) NA.	There was more sickness absence for ACT+WDI compared with TAU. Net days with sickness absence were 61 pre-treatment and 17 at 9-month follow-up. Within-group effect sizes for symptoms of depression, anxiety, and stress ranged from −0.06 to 0.70 for the TAU group.
Grossi & Santell (2009)	(1) Sweden (2) Stress	(1) Quasi-experimental (2) *n* = 12 (3) TAU + Group for coping with stress (*n* = 12) (4) 75% (100% sick leave)	(1) OHS. (2) NA. (3) Guidelines from the Swedish Psychiatric Association: Psychoeducation, psychopharmacology, psychotherapy, relaxation, physical exercise, workplace rehabilitation. (4) 12-week program for intervention group. Not specified for TAU.	40% had returned to work (same for both conditions) at 5-year follow-up. Depression decreased in both conditions.
Hees et al. (2013)	(1) The Netherlands (2) Depression	(1) RCT (2) *n* = 39 (3) Adjuvant occupational therapy (OT, *n* = 78) (4) 56% (for more than 3 months)	(1) Psychiatric outpatient at a university clinic. (2) Psychiatric residents. (3) APA guidelines: Psychoeducation, supportive, CBT, pharmacological. (4) 18 sessions with OT. Not specified for TAU.	The groups did not differ in their overall work participation, but the intervention group showed greater improvement in depression symptoms at 18-month follow-up. 89% with partial RTW and 56% with full RTW. Median days to partial RTW: 166 (67–350) and 405 (189–613) for full RTW.
Kausto et al. (2022)	(1) Finland (2) CMD	(1) Register (2) *n* = 10,436 (3) NA (4) 100%	(1) Specialist clinic. (2) NA. (3) Statutory rehabilitative psychotherapy: psychodynamic, CBT, solution-focused, integrative. (4) 3.01 years.	TAU was associated with a decline in depression-related or anxiety-related work disability. Mental health-related work disability months (0 to 12) decreased from 1.34 to 1.07 months per year.
Rebergen et al. (2009)	(1) The Netherlands (2) CMD	(1) RCT (2) *n* = 115 (3) Guideline-based care (GBC) by occupational physicians (*n* = 125) (4) 100%	(1) OHS (2) “Easy access to psychologist.” (3) NA (4) NA	GBC by OPs did not result in earlier RTW than TAU at 1-year follow-up. 54% had partial RTW and 46% Full RTW. Mean days to partial RTW = 50.6 (78.4), full RTW = 104 (81–127).
Schene et al. (2007)	(1) The Netherlands (2) Depression	(1) RCT (2) *n* = 24 (3) Adjuvant occupational therapy + TAU (*n* = 30) (4) 100%	(1) Psychiatric outpatient. (2) Senior psychiatric residents. (3) APA guidelines (assessment, psychoeducation, support, CBT, pharmacological). (4) 6 months for TAU+OT. TAU = 30 min every 2–3 weeks. Intensity and duration were decided by the physicians.	TAU+OT resulted in fewer work-loss days during the first 18 months, but not at 19-, or 42-month follow-up. TAU+OT did not improve depression more than regular TAU. Partial RTW = 41% (vs. 52% for TAU+OT). Mean days to RTW = 299 (vs. 207 for OT + TAU).

CMD, common mental disorders; OHS, Occupational health service; RTW, return-to-work.

Treatments were delivered in different settings. Two took place within primary care, four were described as psychiatric outpatient clinics, two within occupational health services, and one was described as a specialist clinic (statutory rehabilitative psychotherapy in Finland). Descriptions of therapists often lacked; one study used occupational therapists, one Swedish study had a wide range of different mental health care professionals, while two Dutch studies reported the use of psychiatric residents. Another Dutch study reported easy access to a psychologist in their TAU treatment.

Descriptions of treatment were usually ambiguous. Several described a smorgasbord of interventions, often based on a presumption that therapists followed national guidelines. These interventions included psychoeducation, self-help, CBT, integrative treatment, interpersonal therapy, psychopharmacology, acupuncture, relaxation exercises, behavioral treatment, ECT, occupational therapy, psychodynamic therapy, physical activity, supportive therapy, physiotherapy, and workplace rehabilitation. Duration and intensity of treatment were not specified for TAU interventions, however, one study reported that half the sample met with a psychologist for a mean of 4.1 sessions, while the Finish register-based study had a mean of 3.01 years of statutory rehabilitative psychotherapy. One Dutch study specified that TAU consisted of 30 min every 2–3 weeks, but intensity and duration were decided by the physicians.

In the RCTs, two studies favored the intervention, one favored routine care, and three found no difference between conditions. Choice of outcomes differed greatly and included RTW rates (full or partial), number of days until RTW, change in sick leave status, and net days/months of work absence. Regarding partial return to work in the RCTs, the results ranged from 33 to 89% for TAU. For full RTW, the results ranged from 41 to 56% for TAU. Two studies reported mean days until partial RTW with one study finding 51 days (104 days until full return) and the other 299 days. One Dutch study found a median of 166 days until partial RTW and 405 days until full RTW, while another Dutch study found a median of 47 days until partial RTW. In summary, the results varied greatly, as did the choice and timing of the outcome assessment (from 3 months to 5 years after treatment).

## Discussion

In this systematic review, we aimed to summarize and evaluate the effects of clinical representative treatment on RTW among patients with a common mental disorder, who received treatment within public mental health care. We identified 141 potential records, indicating that the field has received considerable research attention. However, when applying the pre-registered inclusion/exclusion criteria based on definitions by Shadish et al. (1997), only one record was retained. This highlights the lack of knowledge in the field and underscores the need for further research to determine whether routine care is associated with reduced sickness absence and increased work participation.

Inclusion criteria were broadened to include studies that only examined one specified CMD group and unmanualized psychotherapy given within an OHS setting, resulting in a total of nine included studies. These nine records shared common features as they ensured that patients with some type of CMD were offered some form of active non-manualized psychotherapy treatment and had RTW or sick leave as an outcome.

Of the nine included studies there were six RCTs, two register-based studies, and one quasi-experimental study. Sample sizes per condition in the RCTs ranged from 24 to 184, and TAU was often compared with TAU plus an additional intervention, thus resulting in uneven amounts of treatment delivered between conditions. Treatments were provided in different settings, but descriptions of therapists often lacked as did descriptions of treatments. Duration and intensity of treatment were usually not specified for TAU but ranged from four sessions to 3 years of psychotherapy. Regarding the effect on RTW, in the RCTs, two studies favored the intervention, one favored routine care, and three found no difference between conditions. Although this could indicate that the effect of TAU was equal to or superior to the main intervention in four of six RCTs, the high variability of TAU limits firm conclusions about the effect on RTW compared to work-directed interventions. Moreover, the choice of outcomes differed greatly as did the timing of the outcome assessment, which ranged from 3 months to 5 years after treatment. In summary, we identified a great variation in what was considered routine treatment in the included studies, and the effect on RTW was unclear.

The identified knowledge gap is particularly striking considering that mental disorders have been noted as the largest contributor to the burden of disease worldwide, with large resources spent on treatment, yet the effectiveness of the treatment most patients receive is rarely evaluated or researched (Vigo et al., 2016). Moreover, as evident from the present review, studies rarely report if the treatment has an effect on sick leave or RTW, which results in a limited understanding of the effect of clinically representative treatments on RTW. This makes it difficult to generalize and examine the quality and effect of routine treatment (Wampold et al., 2011; Kazdin, 2015; Watts et al., 2015).

The present review has several implications for research and practice. At the level of clinics, it is imperative to implement systematic monitoring of routine treatment to study treatment effects and potential moderators of outcomes and RTW. This is particularly vital considering the large number of patients receiving treatment at public mental health care facilities. Given the inconclusive evidence regarding the effect of specific RTW interventions (Pomaki et al., 2011; Dewa et al., 2015; Perski et al., 2017; Axén et al., 2020) and the unclear effect of routine treatment on RTW identified in the present review, routine monitoring of RTW outcomes of clinically representative treatments is needed. Combining self-reporting of functioning (e.g., The Work and Social Adjustment Scale; Mundt et al., 2002) and symptoms (e.g., anxiety and depression symptoms) along with register data for work participation could assist clinicians in making RTW an integral part of the treatment goal.

At the research level, it is essential to conduct more and better studies within routine care, including evaluations of the effect of TAU on work functioning and RTW. The present review highlights the scarce reporting of the content of routine treatment, and future studies should provide clear descriptions of interventions, duration and intensity, and therapist profession. Moreover, in studies where TAU is a control condition, this review highlights the need for clearer definitions of the contents of TAU, which would greatly enhance the ability to generalize findings.

This is the first review on the RTW effects of clinical representative TAU amongst individuals with CMD. Even though TAU is the most common treatment for patients with CMD, only nine records were identified after the broadening of inclusion criteria, which are too few studies to conduct a meta-analysis. The findings highlight the need for more and better research on TAU for CMD including RTW as the primary outcome. Consequently, there is no sufficient evidence to conclude regarding the RTW effect of TAU provided within public mental healthcare for people with CMD.

## Data availability statement

The original contributions presented in the study are included in the article/Supplementary material, further inquiries can be directed to the corresponding author.

## Author contributions

JL, MB, ML, AH, SS, and OH designed the study and the search strategy. JL and MB extracted the studies. JL and OH rated risk of bias. JL wrote the first draft of the manuscript. All authors contributed to revising the manuscript and approved the final manuscript.
