# Supplementary material for: Examination of the knowledge gap of return-to-work outcomes in routine outpatient treatment for common mental disorders: a systematic review
Source: Front Psychol. 2023 Nov 3;14:1167058. doi: 10.3389/fpsyg.2023.1167058 (PMC10655137; doi:10.3389/fpsyg.2023.1167058)
Supplement: Supplementary file 1 [file Data_Sheet_1.pdf]

Supplements to:

Examination of the knowledge gap of return-to-work outcomes in routine outpatient treatment for common mental disorders: a systematic review

S1 *PRISMA guidelines.*

S2 *Search strategy in PubMED, PsycINFO, EMBASE, and SveMED+.*

S3 *References to full-text records excluded, with reasons.*

S1 *PRISMA 2020 Checklist*

Section and Topic	Item #	Checklist item	Location where item is reported
**TITLE**			
Title	1	Identify the report as a systematic review.	1
**ABSTRACT**			
Abstract	2	See the PRISMA 2020 for Abstracts checklist.	1
**INTRODUCTION**			
Rationale	3	Describe the rationale for the review in the context of existing knowledge.	2
Objectives	4	Provide an explicit statement of the objective(s) or question(s) the review addresses.	1
**METHODS**			
Eligibility criteria	5	Specify the inclusion and exclusion criteria for the review and how studies were grouped for the syntheses.	3
Information sources	6	Specify all databases, registers, websites, organisations, reference lists and other sources searched or consulted to identify studies. Specify the date when each source was last searched or consulted.	3
Search strategy	7	Present the full search strategies for all databases, registers and websites, including any filters and limits used.	S2
Selection process	8	Specify the methods used to decide whether a study met the inclusion criteria of the review, including how many reviewers screened each record and each report retrieved, whether they worked independently, and if applicable, details of automation tools used in the process.	3
Data collection process	9	Specify the methods used to collect data from reports, including how many reviewers collected data from each report, whether they worked independently, any processes for obtaining or confirming data from study investigators, and if applicable, details of automation tools used in the process.	3
Data items	10a	List and define all outcomes for which data were sought. Specify whether all results that were compatible with each outcome domain in each study were sought (e.g. for all measures, time points, analyses), and if not, the methods used to decide which results to collect.	4
	10b	List and define all other variables for which data were sought (e.g. participant and intervention characteristics, funding sources). Describe any assumptions made about any missing or unclear information.	4
Study risk of bias assessment	11	Specify the methods used to assess risk of bias in the included studies, including details of the tool(s) used, how many reviewers assessed each study and whether they worked independently, and if applicable, details of automation tools used in the process.	3
Effect measures	12	Specify for each outcome the effect measure(s) (e.g. risk ratio, mean difference) used in the synthesis or presentation of results.	5
Synthesis methods	13a	Describe the processes used to decide which studies were eligible for each synthesis (e.g. tabulating the study intervention characteristics and comparing against the planned groups for each synthesis (item #5)).	3
	13b	Describe any methods required to prepare the data for presentation or synthesis, such as handling of missing summary statistics, or data conversions.	3
	13c	Describe any methods used to tabulate or visually display results of individual studies and syntheses.	3
	13d	Describe any methods used to synthesize results and provide a rationale for the choice(s). If meta-analysis was performed, describe the model(s), method(s) to identify the presence and extent of statistical heterogeneity, and software package(s) used.	3

Section and Topic	Item #	Checklist item	Location where item is reported
	13e	Describe any methods used to explore possible causes of heterogeneity among study results (e.g. subgroup analysis, meta-regression).	3
	13f	Describe any sensitivity analyses conducted to assess robustness of the synthesized results.	3
Reporting bias assessment	14	Describe any methods used to assess risk of bias due to missing results in a synthesis (arising from reporting biases).	3
Certainty assessment	15	Describe any methods used to assess certainty (or confidence) in the body of evidence for an outcome.	3
**RESULTS**			
Study selection	16a	Describe the results of the search and selection process, from the number of records identified in the search to the number of studies included in the review, ideally using a flow diagram.	3
	16b	Cite studies that might appear to meet the inclusion criteria, but which were excluded, and explain why they were excluded.	S3
Study characteristics	17	Cite each included study and present its characteristics.	6
Risk of bias in studies	18	Present assessments of risk of bias for each included study.	5
Results of individual studies	19	For all outcomes, present, for each study: (a) summary statistics for each group (where appropriate) and (b) an effect estimate and its precision (e.g. confidence/credible interval), ideally using structured tables or plots.	6
Results of syntheses	20a	For each synthesis, briefly summarise the characteristics and risk of bias among contributing studies.	6
	20b	Present results of all statistical syntheses conducted. If meta-analysis was done, present for each the summary estimate and its precision (e.g. confidence/credible interval) and measures of statistical heterogeneity. If comparing groups, describe the direction of the effect.	5
	20c	Present results of all investigations of possible causes of heterogeneity among study results.	5
	20d	Present results of all sensitivity analyses conducted to assess the robustness of the synthesized results.	5
Reporting biases	21	Present assessments of risk of bias due to missing results (arising from reporting biases) for each synthesis assessed.	5
Certainty of evidence	22	Present assessments of certainty (or confidence) in the body of evidence for each outcome assessed.	5
**DISCUSSION**			
Discussion	23a	Provide a general interpretation of the results in the context of other evidence.	5
	23b	Discuss any limitations of the evidence included in the review.	5
	23c	Discuss any limitations of the review processes used.	8
	23d	Discuss implications of the results for practice, policy, and future research.	8
**OTHER INFORMATION**			

Section and Topic	Item #	Checklist item	Location where item is reported
Registration and protocol	24a	Provide registration information for the review, including register name and registration number, or state that the review was not registered.	1
	24b	Indicate where the review protocol can be accessed, or state that a protocol was not prepared.	8
	24c	Describe and explain any amendments to information provided at registration or in the protocol.	8
Support	25	Describe sources of financial or non-financial support for the review, and the role of the funders or sponsors in the review.	8
Competing interests	26	Declare any competing interests of review authors.	8
Availability of data, code and other materials	27	Report which of the following are publicly available and where they can be found: template data collection forms; data extracted from included studies; data used for all analyses; analytic code; any other materials used in the review.	8

S2 *Search strategy in PubMED, PsycINFO, EMBASE, and SveMED+*

The search strategy was first piloted in PubMed, and then adjusted for mapping terms in PsycINFO, Medline and SveMED+. The search string was made of three main concepts: ‘absenteeism’, ´mental health´, and ´treatment as usual´.

Search strategy in PubMED

((((((((((("Absenteeism"[Mesh]) OR "Sick Leave"[Mesh]) OR "Return to Work/psychology"[Mesh]) OR “Back to work”[Title/Abstract]) OR "sick leave"[Title/Abstract]) OR "sickness absence"[Title/Abstract]) OR "Return to work"[Title/Abstract]) OR "medical leave"[Title/Abstract]) OR "sick listed”[Title/Abstract]) OR "Return to Work"[MAJR]) OR "Sick Leave"[MAJR]) AND(((((((((((((((((((((((((((((("mental disorder*"[Title/Abstract]) OR "mental health*"[Title/Abstract]) OR "psychiatric diagnos*"[Title/Abstract]) OR "mental health"[MeSH]) OR "mental disorders"[MeSH]) OR "mental illness"[Title/Abstract]) OR "functional impairment*"[Title/Abstract]) OR (“common mental disorders”[TITLE/ABSTRACT]) OR “CMD”[TITLE/ABSTRACT]) OR “Anxiety” [TITLE/ABSTRACT]) OR “anxious” [TITLE/ABSTRACT]) OR ”Depress*”[TITLE/ABSTRACT]) OR “dysthym*”[TITLE/ABSTRACT]) OR “generalized anxiety disorder” [TITLE/ABSTRACT]) OR “panic disorder” [TITLE/ABSTRACT]) OR phobia [TITLE/ABSTRACT]) OR “social anxiety disorder” [TITLE/ABSTRACT]) OR “obsessive-compulsive disorder” [TITLE/ABSTRACT]) OR "Psychological stress" [TITLE/ABSTRACT]) OR "Life Stress" [TITLE/ABSTRACT]) OR "Psychologic Stress" [TITLE/ABSTRACT]) OR "Mental suffering" [TITLE/ABSTRACT]) OR “Anguish”[TITLE/ABSTRACT]) OR "Emotional stress”[TITLE/ABSTRACT]) OR “affective*”[TITLE/ABSTRACT]) OR “mood disorder*”[TITLE/ABSTRACT]) OR “Stress” [TITLE/ABSTRACT]) OR “burnout” [TITLE/ABSTRACT]) OR “adjustment disorder*”[TITLE/ABSTRACT]) OR “insomnia*”[TITLE/ABSTRACT]) OR “emotional” [TITLE/ABSTRACT]) OR “distress” [TITLE/ABSTRACT]) AND ((((((((((((("psychotherapy"[MeSH]) OR “psychological intervention” [Title/Abstract]) OR "counsel*"[Title/Abstract]) OR "counseling"[MeSH Terms]) OR " care as usual"[Title/Abstract]) OR "standard treatment*" [Title/Abstract]) OR "standard care*"[Title/Abstract]) OR "Treatment-as-usual"[Title/Abstract]) OR "(tau)"[Title/Abstract]) OR “(cau)"[Title/Abstract]) OR “care-as-usual"[Title/Abstract]) OR “routine care"[Title/Abstract]) OR "routine*"[Title/Abstract])

Search strategy in PsycINFO with an Ovid interface

1. back to work.tw.
2. sick leave.tw.
3. sickness absence.tw.
4. return to work.tw.
5. medical leave.tw.
6. sick listed.tw.
7. return to work.mh.
8. absenteeism.mh.
9. sick leave.mh.
10. "mental disorder*".tw.
11. "mental health*".tw.
12. "psychiatric diagnos*".tw.
13. "mental illness".tw.
14. "functional impairment*".tw.
15. common mental disorders.tw.
16. CMD.tw.
17. anxiety.tw.
18. anxious.tw.
19. depress*.tw.
20. dysthym*.tw.
21. generalized anxiety disorder.tw.
22. panic disorder.tw.
23. phobia.tw.
24. social anxiety disorder.tw.
25. obsessive-compulsive disorder.tw.
26. psychological stress.tw.
27. life stress.tw.
28. psychologic Stress.tw.
29. anguish.tw.
30. mental suffering.tw.
31. emotional stress.tw.
32. affective*.tw.

33. mood disorder*.tw.
34. burnout.tw.
35. adjustment disorder*.tw.
36. insomnia*.tw.
37. emotional.tw.
38. distress.tw.
39. mental health.mh.
40. mental disorders.mh.
41. psychological intervention.tw.
42. counsel*.tw.
43. care as usual.tw.
44. standard treatment*.tw.
45. standard care*.tw.
46. treatment as usual.tw.
47. tau.tw.
48. cau.tw.
49. routine*.tw.
50. routine care.tw.
51. psychotherapy.mh.
52. counseling.mh.
53. 1 or 2 or 3 or 4 or 5 or 6 or 7 or 8 or 9
54. 10 or 11 or 12 or 13 or 14 or 15 or 16 or 17 or 18 or 19 or 20 or 21 or 22 or 23 or 24 or 25 or 26 or 27 or 28 or 29 or 30 or 31 or 32 or 33 or 34 or 35 or 36 or 37 or 38 or 39 or 40
55. 41 or 42 or 43 or 44 or 45 or 46 or 47 or 48 or 49 or 50 or 51 or 52
56. 49 and 52 and 55

Search strategy in EMBASE with an Ovid interface

1. return to work.mp.
2. absenteeism.mp.
3. sick leave.mp.
4. back to work.tw.
5. sick leave.tw.
6. sickness absence.tw.
7. return to work.tw.
8. medical leave.tw.
9. sick listed.tw.
10. 1 or 2 or 3 or 4 or 5 or 6 or 7 or 8 or 9
11. mental health.mp.
12. mental disorders.mp.
13. "mental disorder*".tw.
14. "mental health*".tw.
15. "psychiatric diagnos*".tw.
16. "mental illness".tw.
17. "functional impairment*".tw.
18. common mental disorders.tw.
19. CMD.tw.
20. anxiety.tw.
21. anxious.tw.
22. depress*.tw.
23. dysthym*.tw.
24. generalized anxiety disorder.tw.
25. panic disorder.tw.
26. phobia.tw.
27. social anxiety disorder.tw.
28. obsessive-compulsive disorder.tw.
29. psychological stress.tw.
30. life Stress.tw.
31. psychologic Stress.tw.
32. anguish.tw.
33. mental suffering.tw.
34. emotional stress.tw.
35. affective*.tw.
36. mood disorder*.tw.
37. burnout.tw.
38. adjustment disorder*.tw.
39. insomnia*.tw.
40. emotional.tw.
41. distress.tw.
42. 11 or 12 or 13 or 14 or 15 or 16 or 17 or 18 or 19 or 20 or 21 or 22 or 23 or 24 or 25 or 26 or 27 or 28 or 29 or 30 or 31 or 32 or 33 or 34 or 35 or 36 or 37 or 38 or 39 or 40 or 41
43. psychotherapy.mp.
44. psychological intervention.tw.
45. counsel*.tw.
46. care as usual.tw.
47. standard treatment*.tw.
48. standard care*.tw.
49. treatment as usual.tw.
50. tau.tw.
51. cau.tw.
52. routine*.tw.
53. routine care.tw.
54. 43 or 44 or 45 or 46 or 47 or 48 or 49 or 50 or 51 or 52 or 53
55. 10 and 42 and 54

Search strategy in SveMed+

1. return to work
2. absenteeism
3. sick leave
4. back to work
5. sick leave
6. sickness absence
7. return to work
8. medical leave
9. sick listed
10. 1 or 2 or 3 or 4 or 5 or 6 or 7 or 8 or 9
11. mental health
12. mental disorders
13. "mental disorder*"
14. "mental health*"
15. "psychiatric diagnos*"
16. "mental illness"
17. "functional impairment*"
18. common mental disorders
19. CMD
20. anxiety
21. anxious
22. depress*
23. dysthym
24. generalized anxiety disorder
25. panic disorder
26. phobia
27. social anxiety disorder
28. obsessive-compulsive disorder
29. psychological stress
30. life stress
31. mental suffering
32. anguish
33. emotional stress
34. affective*
35. mood disorder*
36. stress
37. burnout
38. adjustment disorder*
39. insomnia*
40. emotional
41. distress
42. 11 or 12 or 13 or 14 or 15 or 16 or 17 or 18 or 19 or 20 or 21 or 22 or 23 or 24 or 25 or 26 or 27 or 28 or 29 or 30 or 31 or 32 or 33 or 34 or 35 or 36 or 37 or 38 or 39 or 40 or 41
43. psychotherapy
44. psychological intervention
45. counsel*
46. care as usual
47. standard treatment*
48. standard care*
49. treatment as usual
50. (tau)
51. (cau)
52. routine*
53. routine care
54. 43 or 44 or 45 or 46 or 47 or 48 or 49 or 50 or 51 or 52 or 53
55. 10 and 42 and 54

S3 *References to full-text records excluded, with reasons.*

Note: A record may be excluded based on failing more than one inclusion criterion but is classified according to the first criterion failed in our list of inclusion criteria.

Not CMD

Aasdahl, L., Pape, K., Vasseljen, O., Johnsen, R., & Fimland, M. S. (2019). Improved Expectations About Length of Sick Leave During Occupational Rehabilitation Is Associated with Increased Work Participation. *Journal of Occupational Rehabilitation*, *29*(3), 475–482. https://doi.org/10.1007/s10926-018-9808-4

Aasdahl, L., Pape, K., Vasseljen, O., Johnsen, R., Gismervik, S., Halsteinli, V., Fleten, N., Nielsen, C. V., & Fimland, M. S. (2018). Effect of Inpatient Multicomponent Occupational Rehabilitation Versus Less Comprehensive Outpatient Rehabilitation on Sickness Absence in Persons with Musculoskeletal- or Mental Health Disorders: A Randomized Clinical Trial. *J Occup Rehabil*, *28*(1), 170–179. https://doi.org/10.1007/s10926-017-9708-z

Adler, D. A., Lerner, D., Visco, Z. L., Greenhill, A., Chang, H., Cymerman, E., Azocar, F., & Rogers, W. H. (2015). Improving work outcomes of dysthymia (persistent depressive disorder) in an employed population. *General Hospital Psychiatry*, *37*(4), 352–359. https://doi.org/10.1016/j.genhosppsych.2015.04.001

Allen, H. (2008). Using routinely collected data to augment the management of health and productivity loss. *J Occup Environ Med*, *50*(6), 615–632. https://doi.org/10.1097/JOM.0b031e31817b610c

Arends, I., Bruinvels, D. J., Rebergen, D. S., Nieuwenhuijsen, K., Madan, I., Neumeyer-Gromen, A., Bültmann, U., & Verbeek, J. H. (2012). Interventions to facilitate return to work in adults with adjustment disorders. *Cochrane Database Syst Rev*, *12*, Cd006389. https://doi.org/10.1002/14651858.CD006389.pub2

Baka, A., Van der Zweerde, T., Bosmans, J., Van Straten, A., & Lancee, J. (2019). PMH31 Cost-effectiveness of guided internet-delivered cognitive behavioral therapy in comparison with care-as-usual for patients with insomnia in general practice. *Value in Health*, *22*, S686. https://doi.org/10.1016/j.jval.2019.09.1505

Berglund, E., Anderzén, I., Andersén, Å., Carlsson, L., Gustavsson, C., Wallman, T., & Lytsy, P. (2018). Multidisciplinary Intervention and Acceptance and Commitment Therapy for Return-to-Work and Increased Employability among Patients with Mental Illness and/or Chronic Pain: A Randomized Controlled Trial. *Int J Environ Res Public Health*, *15*(11). https://doi.org/10.3390/ijerph15112424

Björkelund, C., Svenningsson, I., Westman, J., Petersson, E. L., Hange, D., Holst, A., Wallin, L., & Udo, C. (2019, November). Vårdsamordnare för depression – effektivt grepp i primärvården—Gav friskare patienter och hälsoekonomiska vinster [Effects of a care manager organization for care of people with mild-moderate depression in Swedish primary care]. *Lakartidningen*, *116*(FSH3).

Brouwers, E. P., de Bruijne, M. C., Terluin, B., Tiemens, B. G., & Verhaak, P. F. (2007). Cost-effectiveness of an activating intervention by social workers for patients with minor mental disorders on sick leave: A randomized controlled trial. *Eur J Public Health*, *17*(2), 214–220. https://doi.org/10.1093/eurpub/ckl099

Carlsson, L., Englund, L., Hallqvist, J., & Wallman, T. (2013). Early multidisciplinary assessment was associated with longer periods of sick leave: A randomized controlled trial in a primary health care centre. *Scandinavian Journal of Primary Health Care*, *31*(3), 141–146. https://doi.org/10.3109/02813432.2013.811943

Corazon, S. S., Nyed, P. K., Sidenius, U., Poulsen, D. V., & Stigsdotter, U. K. (2018). A Long-Term Follow-Up of the Efficacy of Nature-Based Therapy for Adults Suffering from Stress-Related Illnesses on Levels of Healthcare Consumption and Sick-Leave Absence: A Randomized Controlled Trial. *Int J Environ Res Public Health*, *15*(1). https://doi.org/10.3390/ijerph15010137

Dalgaard, V. L., Aschbacher, K., Andersen, J. H., Glasscock, D. J., Willert, M. V., Carstensen, O., & Biering, K. (2017). Return to work after work-related stress: A randomized controlled trial of a work-focused cognitive behavioral intervention. *Scand J Work Environ Health*, *43*(5), 436–446. https://doi.org/10.5271/sjweh.3655

De Vente, W., Kamphuis, J. H., Emmelkamp, P. M. G., & Blonk, R. W. B. (2008). Individual and group cognitive-behavioral treatment for work-related stress complaints and sickness absence: A randomized controlled trial. *Journal of Occupational Health Psychology*, *13*(3), 214–231. https://doi.org/10.1037/1076-8998.13.3.214

Drake, R. E., Frey, W., Bond, G. R., Goldman, H. H., Salkever, D., Miller, A., Moore, T. A., Riley, J., Karakus, M., & Milfort, R. (2013). Assisting social security disability insurance beneficiaries with schizophrenia, bipolar disorder, or major depression in returning to work. *The American Journal of Psychiatry*, *170*(12), 1433–1441. https://doi.org/10.1176/appi.ajp.2013.13020214

Eklund, M. (2015). Exploring quality of life as an intervention outcome among women with stress-related disorders participating in work rehabilitation. *Patient Relat Outcome Meas*, *6*, 1–7. https://doi.org/10.2147/PROM.S74498

Eklund, M. (2017). Minor long-term effects 3-4 years after the ReDOTM intervention for women with stress-related disorders: A focus on sick leave rate, everyday occupations and well-being.

Work: Journal of Prevention, Assessment & Rehabilitation, *58*(4), 527–536. https://doi.org/10.3233/WOR-172639

Eklund, M., & Erlandsson, L. K. (2014). Women's perceptions of everyday occupations: Outcomes of the Redesigning Daily Occupations (ReDO) programme. *Scand J Occup Ther*, *21*(5), 359–367. https://doi.org/10.3109/11038128.2014.922611

Eklund, M., & Erlandsson, L.-K. (2011). Return to work outcomes of the Redesigning Daily Occupations (ReDO) program for women with stress-related disorders–a comparative study. *Women & Health*, *51*(7), 676–692. https://doi.org/10.1080/03630242.2011.618215

Eriksson, M. C. M., Kivi, M., Hange, D., Petersson, E. L., Ariai, N., Häggblad, P., Ågren, H., Spak, F., Lindblad, U., Johansson, B., & Björkelund, C. (2017). Long-term effects of Internet-delivered cognitive behavioral therapy for depression in primary care—The PRIM-NET controlled trial. *Scand J Prim Health Care*, *35*(2), 126–136. https://doi.org/10.1080/02813432.2017.1333299

Folke, F., Parling, T., & Melin, L. (2012). Acceptance and commitment therapy for depression: A preliminary randomized clinical trial for unemployed on long-term sick leave. *Cognitive and Behavioral Practice*, *19*(4), 583–594. https://doi.org/10.1016/j.cbpra.2012.01.002

Geraedts, A. S., Kleiboer, A. M., Twisk, J., Wiezer, N. M., van Mechelen, W., & Cuijpers, P. (2014). Long-term results of a Web-based guided self-help intervention for employees with depressive symptoms: Randomized controlled trial. *Journal of Medical Internet Research*, *16*(7), 14–28. https://doi.org/10.2196/jmir.3539

Geraedts, A. S., Kleiboer, A. M., Wiezer, N. M., van Mechelen, W., & Cuijpers, P. (2014). Short-term effects of a web-based guided self-help intervention for employees with depressive symptoms: Randomized controlled trial. *Journal of Medical Internet Research*, *16*(5), 3–17. https://doi.org/10.2196/jmir.3185

Gjengedal, R. H., Reme, S. E., Hagen, K. B., Lagerfeld, S., Blonk, R. W. B., Sandin, K., Berge, T., & Hjemdal, O. (2020). Work-focused therapy for common mental disorders: A naturalistic study comparing an intervention group with a waitlist control group. *Work (Reading, Mass.)*, *66*(3), 657–667. https://doi.org/10.3233/WOR-203208

Glasscock, D. J., Carstensen, O., & Dalgaard, V. L. (2018). Recovery from work-related stress: A randomized controlled trial of a stress management intervention in a clinical sample. *Int Arch Occup Environ Health*, *91*(6), 675–687. https://doi.org/10.1007/s00420-018-1314-7

Goorden, M., Huijbregts, K. L. M., Van Marwijk, H. W. J., Beekman, A. T. F., Van Der Feltz-Cornelis, C. M., & Hakkaart-Van Roijen, L. (2015). Cost-utility of collaborative care for major depressive disorder in primary care in the Netherlands. *Journal of Mental Health Policy and Economics*, *18*, S16–S17. https://doi.org/10.1016/j.jpsychores.2015.06.006

Grensman, A., Acharya, B. D., Wändell, P., Nilsson, G. H., Falkenberg, T., Sundin, Ö., & Werner, S. (2018). Effect of traditional yoga, mindfulness-based cognitive therapy, and cognitive behavioral therapy, on health related quality of life: A randomized controlled trial on patients on sick leave because of burnout. *BMC Complement Altern Med*, *18*(1), 80. https://doi.org/10.1186/s12906-018-2141-9

Grime, P. R. (2004). Computerized cognitive behavioural therapy at work: A randomized controlled trial in employees with recent stress-related absenteeism. *Occup Med (Lond)*, *54*(5), 353–359. https://doi.org/10.1093/occmed/kqh077

Hansen, K. B., Haugstad, G. K., Grenness, S., Halck, B. E., Opheim, A., & Haugstad, T. S. (2016). Cognitive and somatic intervention – a non randomized clinical trial. *Tidsskrift for Norsk Psykologforening*, *53*(11), 904–911.

Hitt, D., Tahir, T., Davies, L., Delahay, J., & Kelson, M. (2018). The clinical effectiveness of a cognitive behavioural therapy intervention in a work setting: A 5-year retrospective analysis of outcomes. *Journal of Research in Nursing*, *23*(4), 360–372. https://doi.org/10.1177/1744987117745580

Hollinghurst, S., Peters, T. J., Kaur, S., Wiles, N., Lewis, G., & Kessler, D. (2010). Cost-effectiveness of therapist-delivered online cognitive-behavioural therapy for depression: Randomised controlled trial. *Br J Psychiatry*, *197*(4), 297–304. https://doi.org/10.1192/bjp.bp.109.073080

Hultén, A. M., Bjerkeli, P., & Holmgren, K. (2021). Self-reported sick leave following a brief preventive intervention on work-related stress: A randomised controlled trial in primary health care. *BMJ Open*, *11*(3), e041157. https://doi.org/10.1136/bmjopen-2020-041157

Hultin, H., Lindholm, C., & Möller, J. (2012). Is there an association between long-term sick leave and disability pension and unemployment beyond the effect of health status?–a cohort study. *PloS One*, *7*(4). https://doi.org/10.1371/journal.pone.0035614

Hultqvist, J., Bjerkeli, P., Hensing, G., & Holmgren, K. (2021). Does a brief work-stress intervention prevent sick-leave during the following 24 months? A randomized controlled trial in Swedish primary care. *Work (Reading, Mass.)*, *70*(4), 1141–1150. https://dx.doi.org/10.3233/WOR-205029

Isaksson Ro, K. E., Tyssen, R., Gude, T., & Aasland, O. G. (2012). Will sick leave after a counselling intervention prevent later burnout? A 3-year follow-up study of Norwegian doctors. *Scandinavian Journal of Public Health*, *40*(3), 278–285. https://doi.org/10.1177/1403494812443607

Johansson, O., Bjarehed, J., Lundh, L.-G., Andersson, G., & Carlbring, P. (2019). Effectiveness of guided internet-delivered cognitive behavior therapy for depression in routine psychiatry: A randomized controlled trial. *Internet Interventions*, *17*, 100247. https://doi.org/10.1016/j.invent.2019.100247

Kadowaki, Å., Alvunger, A. K., Larsen, H. I., Persdotter, A., Zak, M. S., Johansson, P., & Nystrom, F. H. (2021). Education of the primary health care staff based on acceptance and commitment therapy is associated with reduced sick leave in a prospective controlled trial. *BMC Fam Pract*, *22*(1), 179. https://doi.org/10.1186/s12875-021-01526-5

Kaldo, V., Lundin, A., Hallgren, M., Kraepelien, M., Strid, C., Ekblom, O., Lavebratt, C., Lindefors, N., Ojehagen, A., & Forsell, Y. (2018). Effects of internet-based cognitive behavioural therapy and physical exercise on sick leave and employment in primary care patients with depression: Two subgroup analyses. *Occupational and Environmental Medicine*, *75*(1), 52–58. https://doi.org/10.1136/oemed-2017-104326

Kroger, C., Bode, K., Wunsch, E.-M., Kliem, S., Grocholewski, A., & Finger, F. (2015). Work-related treatment for major depressive disorder and incapacity to work: Preliminary findings of a controlled, matched study. *Journal of Occupational Health Psychology*, *20*(2), 248–258. https://doi.org/10.1037/a0038341

Lander, F., Friche, C., Tornemand, H., Andersen, J. H., & Kirkeskov, L. (2009). Can we enhance the ability to return to work among workers with stress-related disorders? *BMC Public Health*, *9*, 372. https://doi.org/10.1186/1471-2458-9-372

Lepiece, B., Reynaert, C., Jacques, D., & Zdanowicz, N. (2017). Returning to work after a common mental health disorder: A new preoccupation for mental health professionals? *Psychiatria Danubina*, *29*, 262–266. https://hrcak.srce.hr/263824

Leuzinger-Bohleber, M., Stuhr, U., Rüger, B., & Beutel, M. (2003). How to study the 'quality of psychoanalytic treatments' and their long-term effects on patients' well-being: A representative, multi-perspective follow-up study. *Int J Psychoanal*, *84*, 263–290. https://doi.org/10.1516/C387-0AFM-4P34-M4BT

Lexis, M. A. S., Jansen, N. W. H., Van Amelsvoort, L. G. P. M., Van Den Brandt, P. A., Kant, Ij., Huibers, M. J. H., Berkouwer, A., & Ton, G. T. A. (2011). Prevention of long-term sickness absence and major depression in high-risk employees: A randomised controlled trial. *Occupational and Environmental Medicine*, *68*(6), 400–407. https://doi.org/10.1136/oem.2010.057877

Linden, M. (2003). Epidemiology and treatment of depressive disorders. *Zeitschrift fur Psychosomatische Medizin und Psychotherapie*, *49*(4), 333–345. https://doi.org/10.13109/zptm.2003.49.4.333

Linden, M., & Westram, A. (2010). Prescribing a sedative antidepressant for patients at work or on sick leave under conditions of routine care. *Pharmacopsychiatry*, *43*(1), 1–6. https://doi.org/10.1055/s-0029-1231076

Lopez-Garcia-Franco, A., del-Cura-Gonzalez, M. I., Caballero-Martinez, L., Sanz-Cuesta, T., Diaz-Garcia, M. I., Rodriguez-Monje, M. T., Chahua, M., Munoz-Sanchez, I., Serrano-Gonzalez, D.,

Rollan-Llanderas, T., Nieto-Blanco, E., Losada-Cucco, L., Caballero-Martinez, F., Sanz-Garcia, N., Pose-Garcia, B., Jurado-Sueiro, M., Rey, M. L., de Blas Gonzalez, F. G., Abanto, M. A. M., … Ascanio-Duran, C. (2012). Effectiveness of a cognitive behavioral intervention in patients with medically unexplained symptoms: Cluster randomized trial. *BMC Family Practice*, *13*, 35. https://doi.org/10.1186/1471-2296-13-35

Maija, K., & Katri, K. (2019). The moral orders of work and health: A case of sick leave due to burnout. *Sociology of Health & Illness*, *41*(2), 219–233. https://doi.org/10.1111/1467-9566.12816

Mauramo, E., Lahti, J., Lallukka, T., Lahelma, E., Pietiläinen, O., & Rahkonen, O. (2019). Changes in common mental disorders and diagnosis-specific sickness absence: A register-linkage follow-up study among Finnish municipal employees. *Occupational and Environmental Medicine*, *76*(4), 230–235. https://doi.org/10.1136/oemed-2018-105423

Muschalla, B. (2016). Negative work perception not changed in a short work-anxiety-coping group therapy intervention. *Int J Occup Environ Health*, *22*(4), 321–324. https://doi.org/10.1080/10773525.2016.1238663

Mynors-Wallis, L., Davies, I., Gray, A., Barbour, F., & Gath, D. (1997). A randomised controlled trial and cost analysis of problem-solving treatment for emotional disorders given by community nurses in primary care. *Br J Psychiatry*, *170*, 113–119. https://doi.org/10.1192/bjp.170.2.113

Netterstrøm, B., & Bech, P. (2010). Effect of a multidisciplinary stress treatment programme on the return to work rate for persons with work-related stress. A non-randomized controlled study from a stress clinic. *BMC Public Health*, *10*, 658. https://doi.org/10.1186/1471-2458-10-658

Nicosia, G. J., Minewiser, L., & Freger, A. (2019). World Trade Center: A longitudinal case study for treating Post Traumatic Stress Disorder with Emotional Freedom Technique and Eye Movement Desensitization and Reprocessing. *Work*, *63*(2), 199–204. https://doi.org/10.3233/WOR-192921

Nieuwenhuijsen, K., Bultmann, U., Neumeyer-Gromen, A., Verhoeven, A. C., Verbeek, J. H. A. M., & Van Der Feltz-Cornelis, C. M. (2008). Interventions to improve occupational health in depressed people. *Cochrane Database of Systematic Reviews*, *2*, CD006237. https://doi.org/10.1002/14651858.CD006237.pub2

Nordgreen, T., Havik, O. E., Gjestad, R., Andersson, G., & Carlbring, P. (2018). The effectiveness of guided internet-based cognitive behavioral therapy for social anxiety disorder in a routine care setting. *Internet Interventions*, *13*, 24–29. https://doi.org/10.1016/j.invent.2018.05.003

Ohki, Y., Igarashi, Y., & Yamauchi, K. (2020). Re-work Program in Japan-Overview and Outcome of the Program. *Front Psychiatry*, *11*, 616223. https://doi.org/10.3389/fpsyt.2020.616223

Pedersen, P., Søgaard, H. J., Labriola, M., Nohr, E. A., & Jensen, C. (2015). Effectiveness of psychoeducation in reducing sickness absence and improving mental health in individuals at risk of having a mental disorder: A randomised controlled trial. *BMC Public Health*, *15*, 763. https://doi.org/10.1186/s12889-015-2087-5

Persson Asplund, R., Dagoo, J., Fjellstrom, I., Niemi, L., Ljotsson, B., Hansson, K., Zeraati, F., Ziuzina, M., Geraedts, A., Carlbring, P., & Andersson, G. (2018). Internet-based stress management for distressed managers: Results from a randomised controlled trial. *Occupational and Environmental Medicine*, *75*(2), 105–113. https://doi.org/10.1136/oemed-2017-104458

Raya Tena, A., Falder Serna, I., Fernández Linares, E. M., & Casañas Sánchez, R. (2015). Impacto de una intervención grupal psicoeducativa: Para pacientes con depresión leve/moderada en las consultas de atención primaria [Impact of a psychoeducational group intervention for patients with mild/moderate depression in primary care consultations]. *Rev Enferm*, *38*(10), 58–62.

Rutz, W., Walinder, J., Eberhard, G., Holmberg, G., Von Knorring, A. L., Von Knorring, L., Wistedt, B., & Aberg-Wistedt, A. (1989). An educational program on depressive disorders for general practitioners on Gotland: Background and evaluation. *Acta Psychiatrica Scandinavica*, *79*(1), 19–26. https://doi.org/10.1111/j.1600-0447.1989.tb09229.x

Sandahl, C., Lundberg, U., Lindgren, A., Rylander, G., Herlofson, J., Nygren, A., & Asberg, M. (2011). Two forms of group therapy and individual treatment of work-related depression: A one-year follow-up study. *Int J Group Psychother*, *61*(4), 539–555. https://doi.org/10.1521/ijgp.2011.61.4.538

Sandheimer, C., Hedenrud, T., Hensing, G., & Holmgren, K. (2020). Effects of a work stress intervention on healthcare use and treatment compared to treatment as usual: A randomised controlled trial in Swedish primary healthcare. *BMC Family Practice*, *21*(1), 133. https://doi.org/10.1186/s12875-020-01210-0

Shippee, N. D., Shah, N. D., Angstman, K. B., Bruce, S. M., Williams, M. D., Dejesus, R. S., & Wilkinson, J. M. (2013). Impact of collaborative care for depression on clinical, functional, and work outcomes: A practice-based evaluation. *Journal of Ambulatory Care Management*, *36*(1), 13–23. https://doi.org/10.1097/JAC.0b013e318276dc10

Sieurin, L., Josephson, M., & Vingård, E. (2009). Positive and negative consequences of sick leave for the individual, with special focus on part-time sick leave. *Scandinavian Journal of Public Health*, *37*(1), 50–56. https://doi.org/10.1177/1403494808097171

Stein, M. B., Roy-Byrne, P. P., Craske, M. G., Bystritsky, A., Sullivan, G., Pyne, J. M., Katon, W., & Sherbourne, C. D. (2005). Functional impact and health utility of anxiety disorders in primary

care outpatients. *Med Care*, *43*(12), 1164–1170. https://doi.org/10.1097/01.mlr.0000185750.18119.fd

Stenlund, T., Nordin, M., & Järvholm, L. S. (2012). Effects of rehabilitation programmes for patients on long-term sick leave for burnout: A 3-year follow-up of the REST study. *Journal of Rehabilitation Medicine*, *44*(8), 684–690. https://doi.org/10.2340/16501977-1003

Thiart, H., Ebert, D. D., Lehr, D., Nobis, S., Buntrock, C., Berking, M., Smit, F., & Riper, H. (2016). Internet-Based Cognitive Behavioral Therapy for Insomnia: A Health Economic Evaluation. *Sleep*, *39*(10), 1769–1778. https://doi.org/10.5665/sleep.6152

Tuulio-Henriksson, A., Toikka, T., Heino, P., & Laukkala, T. (2017). The capacity to work or study after rehabiltiative psychotherapy-a register-based cohort study. *Archives of Physical Medicine and Rehabilitation*, *98*(10), e115. https://doi.org/10.1016/j.apmr.2017.08.370

Virtanen, M., Pentti, J., Karlsson, H., Korkeila, J., Klaukka, T., Suoyrjo, H., Kivimaki, M., & Vahtera, J. (2009). Sickness absence trends during and after long-term psychotherapy and antidepressant medication among depressive employees. *Psychotherapy and Psychosomatics*, *78*(2), 130–132. https://doi.org/10.1159/000203121

Wahrborg, P., Petersson, I. F., & Grahn, P. (2014). Nature-assisted rehabilitation for reactions to severe stress and/or depression in a rehabilitation garden: Long-term follow-up including comparisons with a matched population-based reference cohort. *Journal of Rehabilitation Medicine : Official Journal of the UEMS European Board of Physical and Rehabilitation Medicine*, *46*(3), 271–276. https://doi.org/10.2340/16501977-1259

Wang, P. S., Simon, G. E., Avorn, J., Azocar, F., Ludman, E. J., McCulloch, J., Petukhova, M. Z., & Kessler, R. C. (2007). Telephone screening, outreach, and care management for depressed workers and impact on clinical and work productivity outcomes: A randomized controlled trial. *Jama*, *298*(12), 1401–1411. https://doi.org/10.1001/jama.298.12.1401

Wikberg, C., Ariai, N., Nejati, S., Bjorkelund, C., Petersson, E. L., & Westman, J. (2018). Effects on work ability, job strain and quality of life of monitoring depression using a self-assessment instrument in recurrent general practitioner consultations: A randomized controlled study. *Work (Reading, Mass.)*, *60*(1), 63–73. https://doi.org/10.3233/WOR-182717

Wikberg, C., Bjorkelund, C., Westman, J., Petersson, E. L., Eggertsen, R., Thorn, J., Larsson, M. E. H., Andre, M., & Agren, H. (2017). Use of a self-rating scale to monitor depression severity in recurrent GP consultations in primary care—Does it really make a difference? A randomised controlled study. *BMC Family Practice*, *18*(1), 6. https://doi.org/10.1186/s12875-016-0578-9

Yaniv, G. (1998). Phobic disorder, psychotherapy, and risk-taking: An economic perspective. *J Health Econ*, *17*(2), 229–243. https://doi.org/10.1016/S0167-6296(97)00026-X

Zettle, R. D. (2005). ACT with Affective Disorders. *A Practical Guide to Acceptance and Commitment Therapy.*, 77–102. https://doi.org/10.1007/978-0-387-23369-7_4

Specific treatment techniques or modalities

Aanesen, M., Haugen, T., & Kiland, C. (2016). Mindfulness as intervention to prevent and reduce sickness absence. Ergoterapeuten (Oslo), 59(5), 42–45.

Aasdahl, L., Vasseljen, O., Gismervik, S., Johnsen, R., & Fimland, M. S. (2021). Two-Year Follow-Up of a Randomized Clinical Trial of Inpatient Multimodal Occupational Rehabilitation Vs Outpatient Acceptance and Commitment Therapy for Sick Listed Workers with Musculoskeletal or Common Mental Disorders. J Occup Rehabil, 31(4), 721–728. https://doi.org/10.1007/s10926-021-09969-4

De Weerd, B. J., Van Dijk, M. K., Van Der Linden, J. N., Roelen, C. A. M., & Verbraak, M. J. P. M. (2016). The effectiveness of a convergence dialogue meeting with the employer in promoting return to work as part of the cognitive-behavioural treatment of common mental disorders: A randomized controlled trial. Work, 54(3), 647–655. https://doi.org/10.3233/WOR-162307

Demou, E., Brown, J., Sanati, K., Kennedy, M., Murray, K., & Macdonald, E. B. (2015). A novel approach to early sickness absence management: The EASY (Early Access to Support for You) way. Work (Reading, Mass.), 53(3), 597–608. https://doi.org/10.3233/WOR-152137

Depression in the workplace is associated with high indirect costs related to absenteeism and impaired performance. (2008). Drugs and Therapy Perspectives, 24(6), 23–26.

Ejeby, K., Savitskij, R., Ost, L.-G., Ekbom, A., Brandt, L., Ramnero, J., Asberg, M., & Backlund, L. G. (2014). Symptom reduction due to psychosocial interventions is not accompanied by a reduction in sick leave: Results from a randomized controlled trial in primary care. Scandinavian Journal of Primary Health Care, 32(2), 67–72. https://doi.org/10.3109/02813432.2014.909163

Finnes, A., Hoch, J. S., Enebrink, P., Dahl, J., Ghaderi, A., Nager, A., & Feldman, I. (2022). Economic evaluation of return-to-work interventions for mental disorder-related sickness absence: Two years follow-up of a randomized clinical trial. Scandinavian Journal of Work, Environment and Health, 48(4), 264–272. https://dx.doi.org/10.5271/sjweh.4012

Garcia-Sancho, J. C. M., Puerto, A. G., Egea, C., Diaz, G., Cascales, M. J. C., & Gil, R. M. E. (2018). Stepped psychological intervention with common mental disorders in primary care. Anales de Psicologia, 34(1), 30–40. https://doi.org/10.6018/analesps.34.1.281491

Hägglund, P., Johansson, P., & Laun, L. (2020). The Impact of CBT on Sick Leave and Health. Evaluation Review, 44(2), 185–217. https://doi.org/10.1177/0193841X20976516

Jansson, I., Gunnarsson, A. B., Björklund, A., Brudin, L., & Perseius, K. I. (2015). Problem-based self-care groups versus cognitive behavioural therapy for persons on sick leave due to common

mental disorders: A randomised controlled study. J Occup Rehabil, 25(1), 127–140. https://doi.org/10.1007/s10926-014-9530-9

Jarl, J., Linder, A., Busch, H., Nyberg, A., & Gerdtham, U.-G. (2020). Heterogeneity in the associations between common mental disorders and labour outcomes—A population study from southern Sweden. BMC Public Health, 20(1), 1285. https://doi.org/10.1186/s12889-020-09348-3

Kendrick, T., Simons, L., Lathlean, J., Mynors-Wallis, L., Gray, A., Rivero-Arias, O., Pickering, R., Harris, S., Gerard, K., & Thompson, C. (2005). A trial of problem-solving by community mental health nurses for anxiety, depression and life difficulties among general practice patients. The CPN-GP study. Health Technology Assessment, 9(37). http://ovidsp.ovid.com/ovidweb.cgi?T=JS&PAGE=reference&D=emed9&NEWS=N&AN=41535990

Knapstad, M., Lervik, L. V., Sæther, S. M. M., Aarø, L. E., & Smith, O. R. F. (2020). Effectiveness of Prompt Mental Health Care, the Norwegian Version of Improving Access to Psychological Therapies: A Randomized Controlled Trial. Psychotherapy and Psychosomatics, 89(2), 90–105. https://doi.org/10.1159/000504453

Knapstad, M., Sæther, S. M. M., Hensing, G., & Smith, O. R. F. (2020). Prompt Mental Health Care (PMHC): Work participation and functional status at 12 months post-treatment. BMC Health Services Research, 20(1), 85. https://doi.org/10.1186/s12913-020-4932-1

Lagerveld, S. E., Blonk, R. W., Brenninkmeijer, V., Wijngaards-de Meij, L., & Schaufeli, W. B. (2012). Work-focused treatment of common mental disorders and return to work: A comparative outcome study. J Occup Health Psychol, 17(2), 220–234. https://doi.org/10.1037/a0027049

Lagerveld, S. E., Brenninkmeijer, V., Blonk, R. W., Twisk, J., & Schaufeli, W. B. (2017). Predictive value of work-related self-efficacy change on RTW for employees with common mental disorders. Occup Environ Med, 74(5), 381–383. https://doi.org/10.1136/oemed-2016-104039

Mauramo, E., Lallukka, T., Lahelma, E., Pietiläinen, O., & Rahkonen, O. (2018). Common Mental Disorders and Sickness Absence: A Register-Linkage Follow-Up Study Among Finnish Municipal Employees. Journal of Occupational and Environmental Medicine, 60(6), 569–575. https://doi.org/10.1097/JOM.0000000000001289

Okamoto, Y., Takanashi, R., Sutoh, C., Domon, Y., Yamada, M., Baba, Y., Aya, C., Yamanouchi, N., Sasaki, H., & Shimizu, E. (2022). Improvement in social anxiety following a return-to-work intervention for patients with depression. Medicine (Baltimore), 101(7), e28845. https://doi.org/10.1097/md.0000000000028845

Øverland, S., Grasdal, A. L., & Reme, S. E. (2018). Long-term effects on income and sickness benefits after work-focused cognitive-behavioural therapy and individual job support: A pragmatic, multicentre, randomised controlled trial. Occup Environ Med, 75(10), 703–708. https://doi.org/10.1136/oemed-2018-105137

Reho, T. T. M., Atkins, S. A., Talola, N., Sumanen, M. P. T., Viljamaa, M., & Uitti, J. (2019). Frequent attenders at risk of disability pension: A longitudinal study combining routine and register data. Scandinavian Journal of Public Health, 1403494819838663. https://doi.org/10.1177/1403494819838663

Reme, S. E., Grasdal, A. L., Løvvik, C., Lie, S. A., & Øverland, S. (2015). Work-focused cognitive-behavioural therapy and individual job support to increase work participation in common mental disorders: A randomised controlled multicentre trial. Occup Environ Med, 72(10), 745–752. https://doi.org/10.1136/oemed-2014-102700

Salomonsson, S., Santoft, F., Lindsäter, E., Ejeby, K., Ljótsson, B., Öst, L. G., Ingvar, M., Lekander, M., & Hedman-Lagerlöf, E. (2017). Cognitive-behavioural therapy and return-to-work intervention for patients on sick leave due to common mental disorders: A randomised controlled trial. Occup Environ Med, 74(12), 905–912. https://doi.org/10.1136/oemed-2017-104342

Sumanen, H., Pietiläinen, O., Lahelma, E., & Rahkonen, O. (2017). 10-year trends of educational differences in long sickness absence due to mental disorders. Journal of Occupational Health, 59(4), 352–355. https://doi.org/10.1539/joh.17-0024-BR

Victor, M., Lau, B., & Ruud, T. (2017). Predictors of return to work among patients in treatment for common mental disorders: A pre-post study. BMC Public Health, 18(1), 27. https://doi.org/10.1186/s12889-017-4581-4

Victor, M., Lau, B., & Ruud, T. (2018). Predictors of Return to Work 6 Months After the End of Treatment in Patients with Common Mental Disorders: A Cohort Study. Journal of Occupational Rehabilitation, 28(3), 548–558. https://doi.org/10.1007/s10926-017-9747-5

Winter, L., Kraft, J., Boss, K., & Kahl, K. G. (2015). Rückkehr ins Erwerbsleben: Ein Arbeitsplatz-bezogenes Modul zur Integration in die kognitiv-behaviorale Therapie bei psychischen Erkrankungen [Return to Work: A Workplace Focused Module to be Integrated in Cognitive Behavioral Therapy]. Psychother Psychosom Med Psychol, 65(8), 321–326. https://doi.org/10.1055/s-0035-1545312

Not RTW outcome

Hange, D., Ariai, N., Björkelund, C., Svenningsson, I., Nejati, S., Petersson, E. L., Augustsson, P., & Skoglund, I. (2021). Associations between antidepressant therapy, work ability, and sick leave for

patients with common mental disorders within a two-year perspective—A longitudinal observational cohort study in Swedish primary care. *Heliyon*, *7*(5), e07116. https://doi.org/10.1016/j.heliyon.2021.e07116

Knudsen, A. K., Harvey, S. B., Mykletun, A., & Øverland, S. (2013). Common mental disorders and long-term sickness absence in a general working population. The Hordaland Health Study. *Acta Psychiatrica Scandinavica*, *127*(4), 287–297. https://doi.org/10.1111/j.1600-0447.2012.01902.x

Meuldijk, D., Carlier, I. V. E., van Vliet, I. M., van Hemert, A. M., Zitman, F. G., & van den Akker-van Marle, M. E. (2015). Economic evaluation of concise cognitive behavioural therapy and/or pharmacotherapy for depressive and anxiety disorders. *Journal of Mental Health Policy and Economics*, *18*(4), 175–183.

Oosterbaan, D. B., Verbraak, M. J. P. M., Terluin, B., Hoogendoorn, A. W., Peyrot, W. J., Muntingh, A., & Van Balkom, A. J. L. M. (2013). Collaborative stepped care v. Care as usual for common mental disorders: 8-month, cluster randomised controlled trial. *British Journal of Psychiatry*, *203*(2), 132–139. https://doi.org/10.1192/bjp.bp.112.125211

Setting

Alonso, S., Marco, J. H., & Andani, J. (2018). Reducing the time until psychotherapy initiation reduces sick leave duration in participants diagnosed with anxiety and mood disorders. *Clin Psychol Psychother*, *25*(1), 138–143. https://doi.org/10.1002/cpp.2134

Arends, I., Bultmann, U., van Rhenen, W., Groen, H., & van der Klink, J. J. L. (2013). Economic evaluation of a problem solving intervention to prevent recurrent sickness absence in workers with common mental disorders. *PLoS ONE*, *8*(8). https://doi.org/10.1371/journal.pone.0071937

Arends, I., Van Der Klink, J. J. L., Bultmann, U., Van Rhenen, W., & De Boer, M. R. (2014). Prevention of recurrent sickness absence in workers with common mental disorders: Results of a cluster-randomised controlled trial. *Occupational and Environmental Medicine*, *71*(1), 21–29. https://doi.org/10.1136/oemed-2013-101412

Dalgaard, L., Eskildsen, A., Carstensen, O., Willert, M. V., Andersen, J. H., & Glasscock, D. J. (2014). Changes in self-reported sleep and cognitive failures: A randomized controlled trial of a stress management intervention. *Scand J Work Environ Health*, *40*(6), 569–581. https://doi.org/10.5271/sjweh.3460

Duijts, S. F. A., Kant, I., van den Brandt, P. A., & Swaen, G. M. H. (2008). Effectiveness of a preventive coaching intervention for employees at risk for sickness absence due to psychosocial health complaints: Results of a randomized controlled trial. *Journal of Occupational and Environmental Medicine*, *50*(7), 765–776. https://doi.org/10.1097/JOM.0b013e3181651584

Ekberg, K., Wåhlin, C., Persson, J., Bernfort, L., & Öberg, B. (2015). Early and Late Return to Work After Sick Leave: Predictors in a Cohort of Sick-Listed Individuals with Common Mental Disorders. *Journal of Occupational Rehabilitation*, *25*(3), 627–637. https://doi.org/10.1007/s10926-015-9570-9

Harkko, J., Sumanen, H., Pietiläinen, O., Piha, K., Mänty, M., Lallukka, T., Rahkonen, O., & Kouvonen, A. (2020). Socioeconomic Differences in Occupational Health Service Utilization and Sickness Absence Due to Mental Disorders: A Register-Based Retrospective Cohort Study. *International Journal of Environmental Research and Public Health*, *17*(6). https://doi.org/10.3390/ijerph17062064

Hellström, L., Hjorthoj, C., Nordentoft, M., Eplov, L. F., Bech, P., & Lindschou, J. (2017). Effect on return to work or education of individual placement and support modified for people with mood and anxiety disorders: Results of a randomised clinical trial. *Occupational and Environmental Medicine*, *74*(10), 717–725. https://doi.org/10.1136/oemed-2016-104248

Keus van de Poll, M., Bergström, G., Jensen, I., Nybergh, L., Kwak, L., Lornudd, C., & Lohela-Karlsson, M. (2020). Cost-Effectiveness of a Problem-Solving Intervention Aimed to Prevent Sickness Absence among Employees with Common Mental Disorders or Occupational Stress. *International Journal of Environmental Research and Public Health*, *17*. https://doi.org/10.3390/ijerph17145234

Keus van de Poll, M., Nybergh, L., Lornudd, C., Hagberg, J., Bodin, L., Kwak, L., Jensen, I., Lohela-Karlsson, M., Torgen, M., & Bergstrom, G. (2020). Preventing sickness absence among employees with common mental disorders or stress-related symptoms at work: A cluster randomised controlled trial of a problem-solving-based intervention conducted by the Occupational Health Services. *Occupational and Environmental Medicine*, *77*(7), 454–461. https://doi.org/10.1136/oemed-2019-106353

Lammerts, L., Vermeulen, S. J., Schaafsma, F. G., van Mechelen, W., & Anema, J. R. (2014). Return to work of workers without a permanent employment contract, sick-listed due to a common mental disorder: Design of a randomised controlled trial. *BMC Public Health*, *14*, 594. https://doi.org/10.1186/1471-2458-14-594

Lohela-Karlsson, M., Kwak, L., Bodin, L., Jensen, I., Nybergh, L., Bergstrom, G., & Torgen, M. (2017). Preventing sickness absenteeism among employees with common mental disorders or stress-related symptoms at work: Design of a cluster randomized controlled trial of a problem-solving based intervention versus care-as-usual conducted at the Occupational Health Services. *BMC Public Health*, *17*(1), 436. https://doi.org/10.1186/s12889-017-4329-1

Lokman, S., Zijlstra-Vlasveld, M., Volker, D., Van Der Feltz-Cornelis, C., & Smit, F. (2015). Return-to-work intervention versus care as usual for sick listed employees with common mental disorders: Trial-based economic evaluation shows promise. *Journal of Mental Health Policy and Economics*, *18*, S26–S27. https://doi.org/10.1136/bmjopen-2017-016348

Marco, J. H., Alonso, S., & Andani, J. (2020). Early intervention with cognitive behavioral therapy reduces sick leave duration in people with adjustment, anxiety and depressive disorders. *Journal of Mental Health (Abingdon, England)*, *29*(3), 247–255. https://doi.org/10.1080/09638237.2018.1521937

Meeuwissen, J. A. C., De Jong, F. J., Van Der Feltz-Cornelis, C. M., Hoedeman, R., & Elfeddali, I. (2007). Randomised controlled trial of a psychiatric consultation model for treatment of common mental disorder in the occupational health setting. *BMC Health Services Research*, *7*, 29. https://doi.org/10.1186/1472-6963-7-29

Momsen, A. H., Stapelfeldt, C. M., Nielsen, C. V., Nielsen, M. B., Aust, B., Rugulies, R., & Jensen, C. (2016). Effects of a randomized controlled intervention trial on return to work and health care utilization after long-term sickness absence. *BMC Public Health*, *16*(1), 1149. https://doi.org/10.1186/s12889-016-3812-4

Nieuwenhuijsen, K., Verbeek, J. H. A. M., De Boer, A. G. E. M., Blonk, R. W. B., & Van Dijk, F. J. H. (2004). Supervisory behaviour as a predictor of return to work in employees absent from work due to mental health problems. *Occupational and Environmental Medicine*, *61*(10), 817–823. https://doi.org/10.1136/oem.2003.009688

Noordik, E., van Dijk, F. J. H., Nieuwenhuijsen, K., van der Klink, J. J., de Boer, M. R., & Geskus, R. B. (2013). Effectiveness of an exposure-based return-to-work program for workers on sick leave due to common mental disorders: A cluster-randomized controlled trial. *Scandinavian Journal of Work, Environment and Health*, *39*(2), 144–154. https://doi.org/0.5271/sjweh.3320

Parsons, V., Juszczyk, D., Gilworth, G., Ntani, G., McCrone, P., Hatch, S., Shannon, R., Henderson, M., Coggon, D., Molokhia, M., Smedley, J., Griffiths, A., Walker-Bone, K., & Madan, I. (2021). A case management occupational health model to facilitate earlier return to work of NHS staff with common mental health disorders: A feasibility study. *Health Technology Assessment*, *25*(12), VII–92. https://dx.doi.org/10.3310/HTA25120

Shiryaeva, O., Victor, M., Lau, B., & Ruud, T. (2019). What are they returning to? Psychosocial work environment as a predictor of returning to work among employees in treatment for common mental disorders: A prospective observational pre-post study. *PLoS ONE*, *14*(4), e0215354. https://doi.org/10.1371/journal.pone.0215354

Suijkerbuijk, Y., & Nieuwenhuijsen, K. (2022). Identification of the return-to-work mode in unemployed workers with mental health issues: A focus group study among occupational health professionals. *Work*. https://doi.org/10.3233/wor-210434

Ubalde-Lopez, M., Arends, I., Almansa, J., Delclos, G. . L., Gimeno, D., & Bültmann, U. (2017). Beyond Return to Work: The Effect of Multimorbidity on Work Functioning Trajectories After Sick Leave due to Common Mental Disorders. *Journal of Occupational Rehabilitation*, *27*(2), 210–217. https://doi.org/10.1007/s10926-016-9647-0

van der Feltz-Cornelis, C. M., Hoedeman, R., de Jong, F. J., Meeuwissen, J. A., Drewes, H. W., van der Laan, N. C., & Adèr, H. J. (2010). Faster return to work after psychiatric consultation for sicklisted employees with common mental disorders compared to care as usual. A randomized clinical trial. *Neuropsychiatr Dis Treat*, *6*, 375–385. https://doi.org/10.2147/ndt.s11832

Volker, D., Zijlstra-Vlasveld, M. C., Anema, J. R., Beekman, A. T., Brouwers, E. P., Emons, W. H., van Lomwel, A. G. C., & van der Feltz-Cornelis, C. M. (2015). Effectiveness of a blended web-based intervention on return to work for sick-listed employees with common mental disorders: Results of a cluster randomized controlled trial. *Journal of Medical Internet Research*, *17*(5), e116. https://doi.org/10.2196/jmir.4097

Volker, D., Zijlstra-Vlasveld, M. C., Brouwers, E. P. M., & van der Feltz-Cornelis, C. M. (2017). Process Evaluation of a Blended Web-Based Intervention on Return to Work for Sick-Listed Employees with Common Mental Health Problems in the Occupational Health Setting. *Journal of Occupational Rehabilitation*, *27*(2), 186–194. https://doi.org/10.1007/s10926-016-9643-4

Wåhlin, C., Ekberg, K., Persson, J., Bernfort, L., & Öberg, B. (2013). Evaluation of self-reported work ability and usefulness of interventions among sick-listed patients. *J Occup Rehabil*, *23*(1), 32–43. https://doi.org/10.1007/s10926-012-9376-y

Language

Holzle, P., Baumbach, A., Mernyi, L., & Hamann, J. (2018). Return to Work: A Psychoeducational Module—An Intervention Study. *Psychiatrische Praxis*, *45*(6), 299–306. https://doi.org/10.1055/s-0043-105775

Jacobi, F., Klose, M., & Wittchen, H. U. (2004). Mental disorders in the community: Healthcare utilization and disability days. *Bundesgesundheitsblatt, Gesundheitsforschung, Gesundheitsschutz*, *47*(8), 736–744. https://doi.org/10.1007/s00103-004-0885-5

Nagata, S., Mishima, N., Ishibashi, S., Miyata, M., Kobayashi, N., Kanazawa, F., Kubota, S., Ueno, T., Mizobe, K., Nitta, Y., Aoki, H., Natsume, T., Ishikawa, T., Takeuchi, K., Fukui, Y., Ito, K., Nomura, S., Takashiba, T., & Fujikawa, H. (1996). Factors influencing reinstatement of the cases

with ‘return to work’ difficulties. *Japanese Journal of Psychosomatic Medicine*, *36*(5), 425–430. https://doi.org/10.15064/jjpm.36.5_425
